# CRISPR-Cas for hepatitis virus: a systematic review and meta-analysis of diagnostic test accuracy studies

**DOI:** 10.3389/fmicb.2025.1509890

**Published:** 2025-03-03

**Authors:** Zhenzhen Pan, Ling Xu, Zihao Fan, Feng Ren

**Affiliations:** Beijing Institute of Hepatology, Beijing Youan Hospital, Capital Medical University, Beijing, China

**Keywords:** CRISPR-Cas, hepatitis virus, nucleic acid testing, diagnostic, meta-analysis

## Abstract

**Background and aims:**

Hepatitis viruses pose a significant global health challenge, necessitating accurate and efficient diagnostic methods. The CRISPR-Cas system, renowned for gene editing, shows potential tool in virus detection. This systematic review and meta-analysis aims to evaluate the diagnostic accuracy of CRISPR-Cas-based tests for hepatitis viruses, aiming to provide evidence for their effectiveness in clinical settings.

**Methods:**

Studies from Web of Science, PubMed, and CNKI were analyzed. A bivariate random-effects model was employed to compute pooled estimates for sensitivity, specificity, and the area under the summary receiver operating characteristic (SROC) curve. Additionally, the methodological quality of the studies was evaluated using the Quality Assessment of Diagnostic Accuracy Studies 2 (QUADAS-2) tool.

**Results:**

Following a rigorous screening process, 14 studies meeting our inclusion criteria were selected from an initial pool of 657 studies. The pooled sensitivity and specificity of the CRISPR-Cas system in hepatitis virus detection showed high sensitivity (0.99, 95% CI: 0.95–1.00) and specificity (0.99, 95% CI: 0.93–1.00) with SROC area 1.00 (95% CI: 0.99–1.00). However, considering the notable heterogeneity among the included studies, subgroup analyses and meta-regression were conducted. These analyses revealed that the type of hepatitis virus detected and the format of the final result presentation could be potential sources of this heterogeneity.

**Conclusion:**

This systematic review and meta-analysis demonstrates the high diagnostic accuracy of CRISPR-Cas system in detecting hepatitis viruses. However, conclusions are limited by study number and quality. Therefore, more high-quality data are still needed to support this conclusion.

## Highlights

- The CRISPR-Cas system demonstrates very high sensitivity and specificity in detecting hepatitis viruses, suggesting it can accurately identify both infected and non-infected individuals.
- There is notable variability among the studies included in the analysis, which may be due to differences in the types of hepatitis viruses being detected and how the test results are presented.
- While the current evidence is promising, the number and quality of existing studies are limited, indicating a need for more high-quality research to confirm the effectiveness of CRISPR-Cas-based tests.

## 1 Introduction

Hepatitis viruses mainly consist of five types: hepatitis A virus (HAV), hepatitis B virus (HBV), hepatitis C virus (HCV), hepatitis D virus (HDV) and hepatitis E virus (HEV) (Lanini et al., 2019). Globally, the number of HBV infection was estimated to be 296 million, with over 95% of cases in low-and middle-income countries. However, only 12–25% of patients have access to diagnosis and antiviral treatment (Yuen et al., 2018; Hsu et al., 2023). Approximately 58 million people worldwide suffer from chronic HCV infection, with 60–80% progressing to chronic hepatitis and potential risks of cirrhosis and liver cancer (Polaris Observatory HCV Collaborators, 2022). Nevertheless, due to the limited diagnostic capability, only 21% of HCV-infected individuals are diagnosed (Martinello et al., 2023; Pietschmann and Brown, 2019). Chronic HDV infection is considered the most severe form of viral hepatitis, and coinfection with HBV accelerates the progression of cirrhosis and hepatocellular carcinoma. Roughly 12 million people are infected with HDV globally, but this estimate remains controversial due to inadequate screening (Negro and Lok, 2023; Stockdale et al., 2020; European Association for the Study of the Liver, 2023). HEV is the leading cause of acute viral hepatitis worldwide, resulting in approximately 20 million infections and 70,000 deaths annually, posing a global public health concern (Aslan and Balaban, 2020; Kamar et al., 2017). In response, the World Health Organization has set a strategy to eliminate viral hepatitis as a public health threat by 2030, focusing on improving the efficiency and coverage of hepatitis virus testing (World Health Organization, 2017). This raises new and higher demands for hepatitis virus detection.

Currently, nucleic acid testing (NAT) is an important index to diagnose hepatitis virus, the most commonly used method of which is quantitative polymerase chain reaction (qPCR) (Pisano et al., 2021; Fu et al., 2023; Manso et al., 2024; Wedemeyer et al., 2023; Germer et al., 2017). Although significant advancements have been made in recent decades, this approach still faces several constraints: (a) it requires technically trained personnel with certified laboratory experience, (b) it often takes a long time to produce results, and (c) the high-sensitivity equipment required for RNA detection is expensive (Gupta et al., 2021). These limitations undermine the versatility of qPCR, especially in areas with limited medical resources. Therefore, cost-effective and highly sensitive NAT methods for hepatitis viruses are crucial for monitoring the occurrence and development of the disease and evaluating its treatment effect.

The CRISPR-Cas system, consisting of clustered regularly interspaced short palindromic repeats (CRISPR) and associated proteins (Cas), functions as a protective immune mechanism in prokaryotes, shielding them from external pathogens. This defense mechanism involves three key steps: first, inserting foreign nucleic acid fragments into the CRISPR sequence; second, initiating crRNA expression through transcription; third, guiding Cas nucleases to the target fragment through mature crRNA and then cleaving the homologous sequence of the invader’s genome (Barrangou and Marraffini, 2014; Hendriks et al., 2020). Due to its simplicity and programmability, the CRISPR-Cas system has rapidly become the preferred gene editing tool (Manghwar et al., 2019). Gootenberg et al. (2017) have established a molecular diagnostic technology platform using CRISPR-Cas system combined with recombinase polymerase amplification (RPA) for NAT, which has attracted widespread attention in the field of molecular diagnostics.

CRISPR-Cas12 and CRISPR-Cas13 exhibit targeted recognition and cleavage of double-stranded DNA (dsDNA) and RNA, respectively (Pickar-Oliver and Gersbach, 2019). When selectively targeting homologous sequences, Cas12 and Cas13 effectors undergo conformational changes, acting as signal amplifiers in NAT, resulting in significantly increased sensitivity. Additionally, this reaction process can be rapidly completed under mild conditions, which is highly beneficial for point-of-care testing (POCT) and large-scale screening of viral hepatitis (Kellner et al., 2019).

Due to the advantages of easy operation, cost-effectiveness, high sensitivity, and specificity in NAT, the use of the CRISPR-Cas system has been applied to the screening of various pathogens, including NAT of hepatitis viruses, but its diagnostic efficacy is still unclear. Therefore, this study aims to provide a brief meta-analysis of research on the diagnosis of hepatitis viruses based on the CRISPR-Cas system to evaluate its diagnostic efficacy (https://www.crd.york.ac.uk/PROSPERO/).

## 2 Methods

This systematic review and meta-analysis were conducted in accordance with the Preferred Reporting Items for Systematic Reviews and Meta-Analyses (PRISMA) guidelines for DTA studies (McInnes et al., 2018). The study protocol was registered in the International Prospective Register of Systematic Reviews (PROSPERO) with registration ID CRD42024559017.

### 2.1 Database sources and searches

Database searches were conducted on Web of Science, PubMed, and CNKI without language restrictions to identify eligible studies on diagnostic test accuracy. Additionally, manual searches were performed on the reference lists of all studies identified by the search strategy. The search strategy included studies published in these databases up to August 1, 2024. Detailed search strategies are provided in the Supplementary File S1.

### 2.2 Inclusion and exclusion criteria

We defined the inclusion criteria as follows: (a) studies that used the CRISPR-Cas system for the detection of hepatitis virus nucleic acid; (b) studies that directly provided data on true positives (TP), false positives (FP), true negatives (TN), and false negatives (FN), or data that could be calculated to obtain these values; (c) studies that used quantitative PCR for nucleic acid on the included samples. However, studies were excluded if they met the following criteria: (a) the number of included samples was less than 15; (b) the study was a case report, review article, or meta-analysis article; (c) the study was a duplicate; (d) the gold standard was unclear or not used.

### 2.3 Study selection and data extraction

After removing duplicate studies, two authors (ZP and LX) independently screened all abstracts and full-texts. Using predetermined inclusion and exclusion criteria, the two authors extracted data from eligible studies. For studies that established multiple CRISPR-related NAT methods, data from different methods were extracted to maximize the amount of data. Disagreements were resolved by a third author (ZF).

Variables extracted from the selected studies included author names, study years, sample sizes, countries where the studies were conducted, types of Cas proteins used in the established methods, types of hepatitis viruses detected, and the presentation of results of the established methods. At the same time, data on TP (true positives), FP (false positives), FN (false negatives), and TN (true negatives) were extracted from the included studies.

### 2.4 Assessment of methodological quality

We used the Quality Assessment of Diagnostic Accuracy Studies 2 (QUADAS-2) tool to evaluate the quality of the included studies (Whiting et al., 2011). This specifically includes the methodological quality of patient selection, index test, reference standard, as well as the flow and timing.

If any question is assessed as high risk, the bias risk for that item is considered high. If an item has two or more questions assessed as low risk, it is considered to have a low bias risk. If an item has two or more questions assessed as unclear, the bias risk for that item is considered unclear. Additionally, the applicability of patient selection, index tests, and reference tests was assessed. All assessments were independently conducted by two authors, and any disagreements were resolved by a third author.

### 2.5 Statistical analysis and data synthesis

By using the “Midas” package in Stata MP 16 software to perform a two-level random effects model analysis on the original research data, we obtained the pooled sensitivity, specificity, and area under the curve (AUC). The estimated values of sensitivity and specificity, along with their 95% confidence intervals (CIs), were presented in forest plot. Additionally, we calculated the summary receiver operating characteristic curve (SROC).

### 2.6 Investigation of heterogeneity

Heterogeneity was indicated by *I*^2^. Furthermore, we explored the potential sources of heterogeneity through subgroup analysis and meta-regression.

## 3 Results

### 3.1 Search results

**Figure 1:**
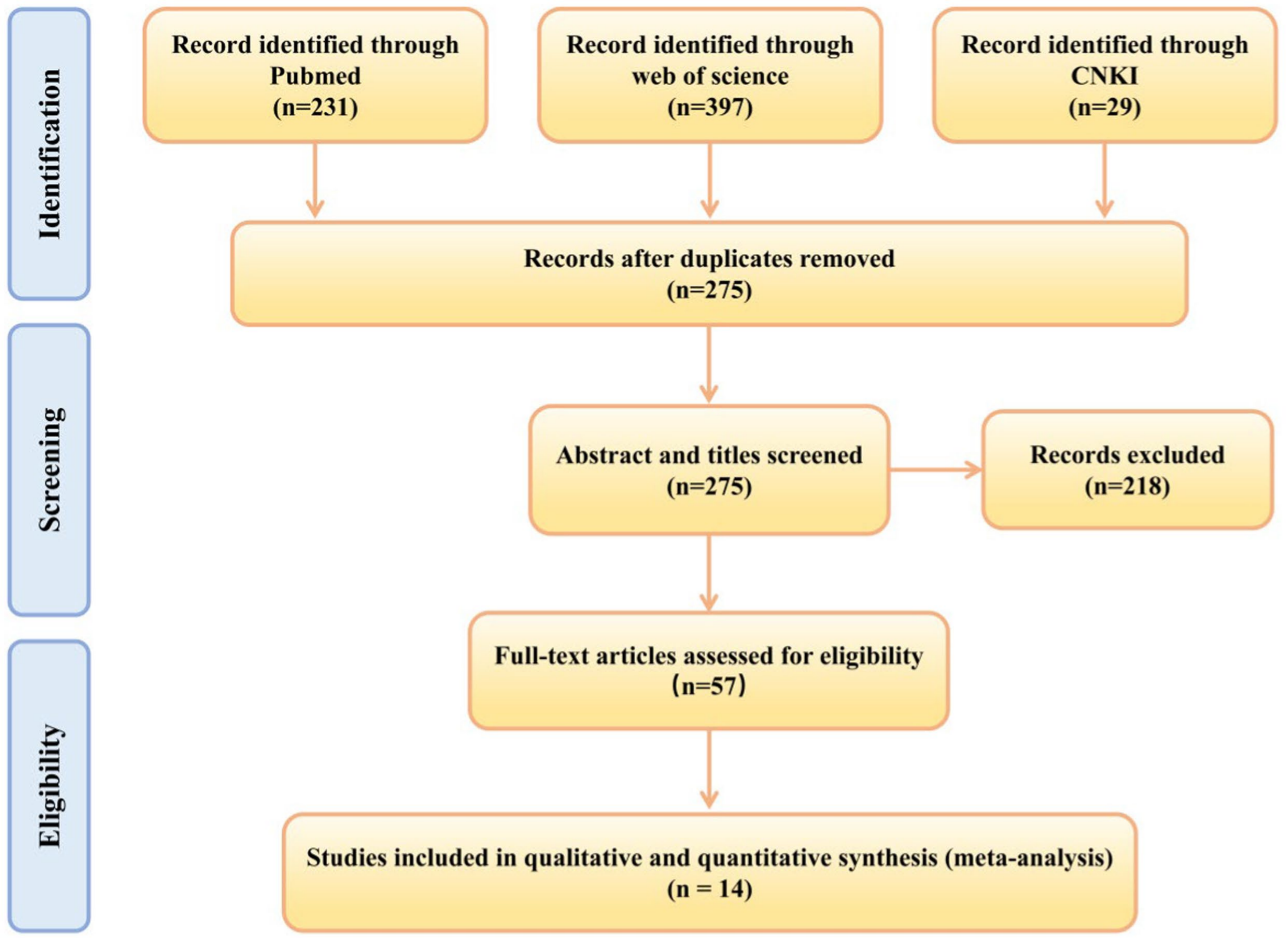
Preferred Reporting Items for Systematic Reviews and Meta-analysis flow diagram: search and study selection process for this review.

After searching three databases, a total of 657 relevant studies were retrieved, and after removing duplicates, there were 275 studies. We screened the 275 studies based on their titles and abstracts, excluding 218 studies. The remaining 57 studies were subject to full-text screening according to the inclusion criteria. Ultimately, this systematic review included 14 studies (Xu et al., 2024; Ho et al., 2023; Yue et al., 2021; He et al., 2024; Nguyen et al., 2023; Li et al., 2023; Kham-Kjing et al., 2022; Ding et al., 2021; Wang et al., 2021; Zhang et al., 2022; Chen et al., 2021; Tian et al., 2023a,b; Tian et al., 2022). A detailed flowchart of the study selection process and various reasons for exclusion is shown in Figure 1.

### 3.2 Characteristics of the included studies

The 14 studies included were published from 2020 to 2024, with 12 studies from China and the remaining two from Thailand and the United States. The sample size of the included studies ranged from 17 to 312, and the types of Cas proteins involved Cas12 (8 studies) and Cas13 (6 studies). The hepatitis viruses detected included HBV, HCV, HDV, and HEV, and the results were presented in two forms: fluorescence and strips. Among them, four studies established both fluorescence and test strip methods. The main features of the included studies using CRISPR for hepatitis virus detection are shown in Table 1.

**Table 1:** The main features of the included studies using CRISPR for hepatitis virus detection.

No.	Author	Country	Year	Sample size	Type of Cas protein	Type of hepatitis viruses	Type(s) of results presented
1	Tian et al. (2023b)	China	2023	144	Cas13a	HDV RNA	Fluorescence
2	Tian et al. (2023a)	China	2023	172	Cas13a	HBV DNA	Fluorescence/strip
3	Li et al. (2023)	China	2023	115	Cas13a	HEV RNA	Fluorescence/strip
4	Zhang et al. (2022)	China	2022	70	Cas13a	HBV cccDNA	Fluorescence
5	Wang et al. (2021)	China	2020	312	Cas13a	HBV DNA	Fluorescence
6	Chen et al. (2021)	China	2021	114	Cas12b	HBV DNA	Strip
7	Kham-Kjing et al. (2022)	Thailand	2022	130	Cas12a	HCV RNA	Fluorescence/strip
8	Ding et al. (2021)	China	2021	73	Cas12a	HBV DNA	Fluorescence/strip
9	Yue et al. (2021)	China	2021	17	Cas12a	HBV DNA	Fluorescence
10	Tian et al. (2022)	China	2022	30	Cas13a	HBV cccDNA	Fluorescence
11	Ho et al. (2023)	China	2023	17	Cas12a	HCV RNA	Fluorescence
12	Xu et al. (2024)	China	2024	236	Cas12b	HBV DNA	Fluorescence
13	He et al. (2024)	China	2024	76	Cas12f1	HBV DNA	Fluorescence
14	Nguyen et al. (2023)	America	2023	80	Cas12b	HCV RNA	Fluorescence

### 3.3 Quality assessments

**Figure 2:**
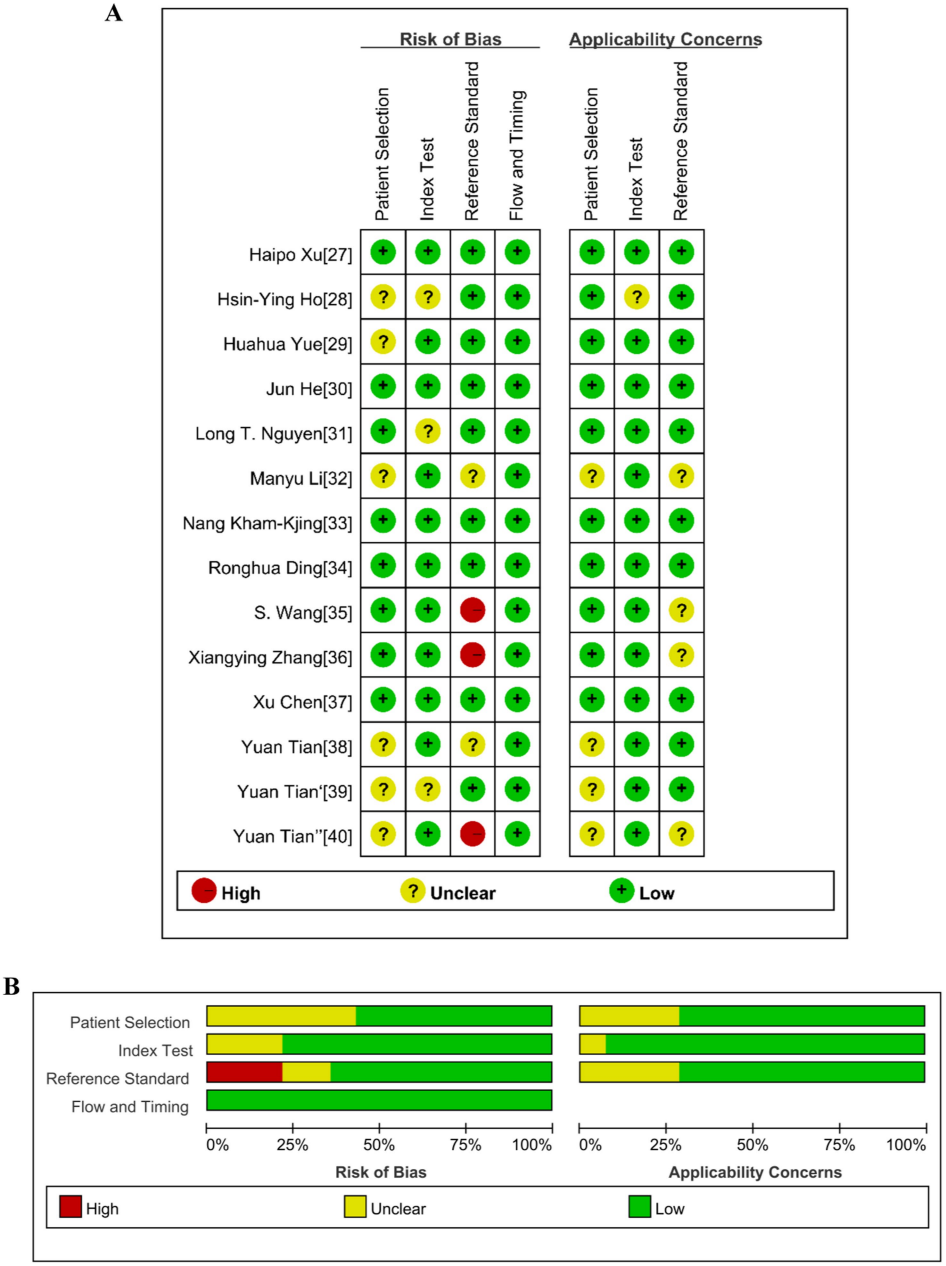
Summary of the risk of bias of the included studies according to the Quality Assessment of Diagnostic Accuracy Studies 2 (QUADAS-2). **(A)** Risk of bias and applicability concerns graph. **(B)** Risk of bias and applicability concerns summary.

Figure 2A displays the individual QUADAS-2 assessment results for the 14 studies included, while Figure 2B presents the summary results. The risk of bias and applicability of all included studies were classified as “high,” “unknown”, and “low.” All 14 studies exhibited a low risk of bias in terms of flow and timing. In the patient selection domain, 42.9% (6/14) of studies had an unclear risk of bias due to the studies not specifying whether continuous or random samples were used, while the remaining studies had a low risk of bias. In the index test domain, 21.4% (3/14) of studies had an “unclear” risk of bias because they did not indicate whether samples were tested without knowledge of the gold standard, while the remaining studies had a low risk of bias. In the reference standard domain, 21.4% (3/14) of studies had a high risk of bias because it was uncertain in these studies whether the reference standard could accurately describe the sample situation. 14.3% (2/14) had an unclear risk of bias, and the remaining 64.3% (9/14) had a low risk of bias. In terms of applicability, 28.6% (4/14) of studies were classified as having an unclear risk in patient selection, while the remaining 71.4% (10/14) were classified as low risk. 7.1% (1/14) of studies were classified as having an unclear risk in the index test, while the remaining 92.9% (13/14) were classified as low risk. 28.6% (4/14) of studies were classified as having an unclear risk in the reference standard, while the remaining 71.4% (10/14) were classified as low risk.

### 3.4 Results of diagnostic test accuracy

**Figure 3:**
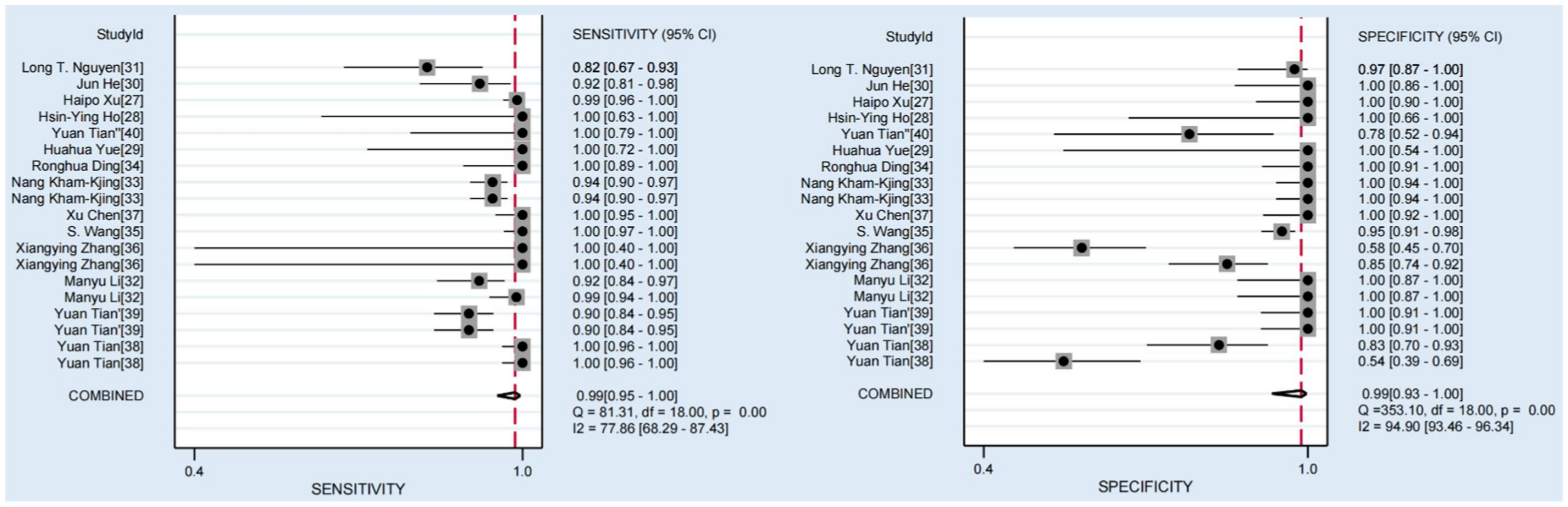
Forest plots of the pooled sensitivity and specificity for CRISPR-Cas system in diagnosis of hepatitis viruses.

**Figure 4:**
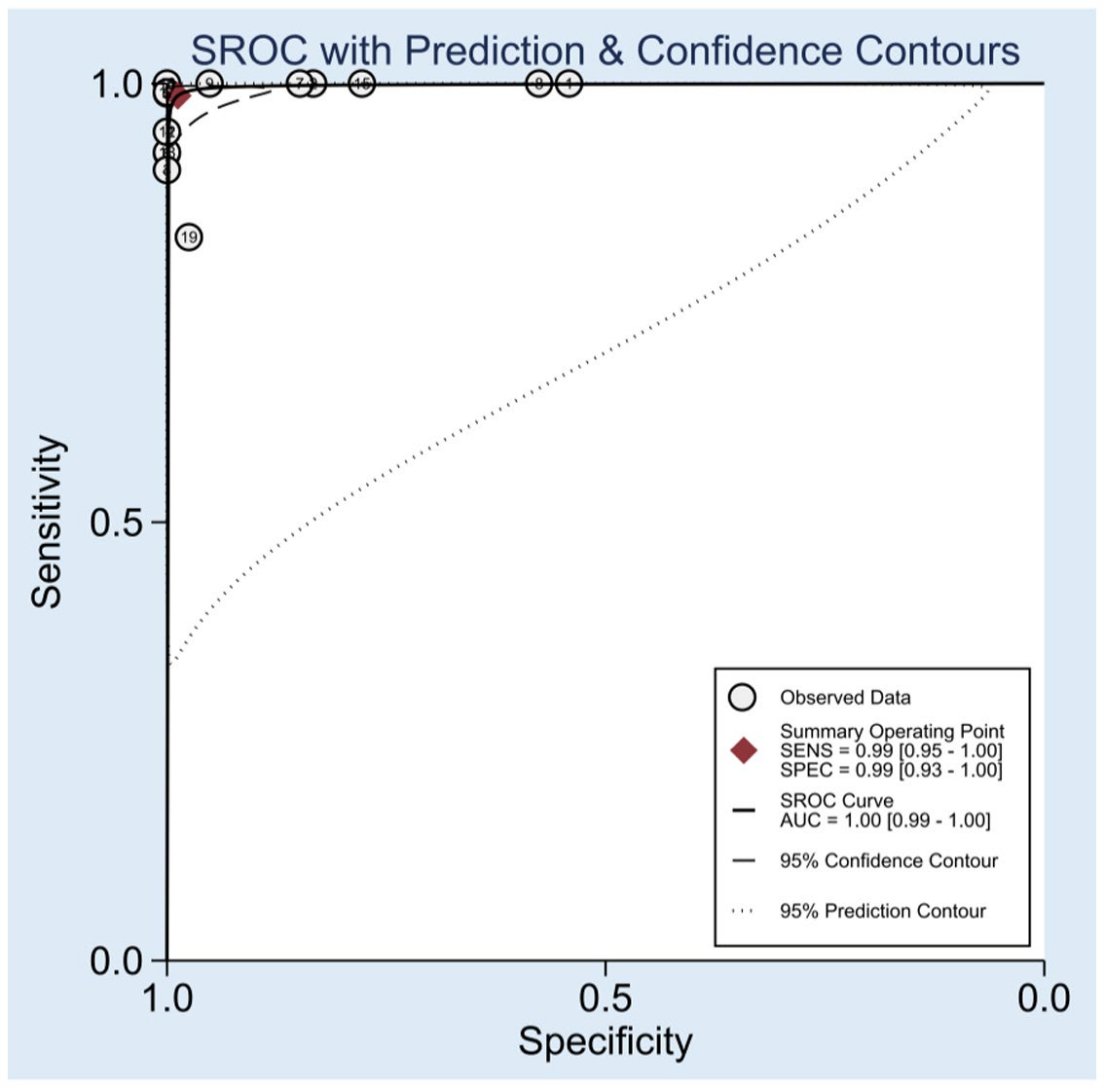
The SROC curves of the CRISPR-Cas system in diagnosis of hepatitis viruses.

Among the 14 studies included, the pooled sensitivity of the CRISPR-Cas system for detecting hepatitis viruses was 0.99 (95% CI 0.95–1.00), and the pooled specificity was 0.99 (95% CI 0.93–1.00). Figure 3 shows the forest plot of sensitivity and specificity for individual studies and the pooled results of the CRISPR-Cas system for detecting hepatitis viruses. The *I*^2^ test indicated significant heterogeneity among the studies. Figure 4 displays the SROC curve, and the area under curve (AUC) was 1.00 (95% CI 0.99–1.00). In addition, the funnel plot showed asymmetry, which was supported by the Egger test (*p* = 0.01) (Supplementary Figure S1).

### 3.5 Subgroup analysis

**Figure 5:**
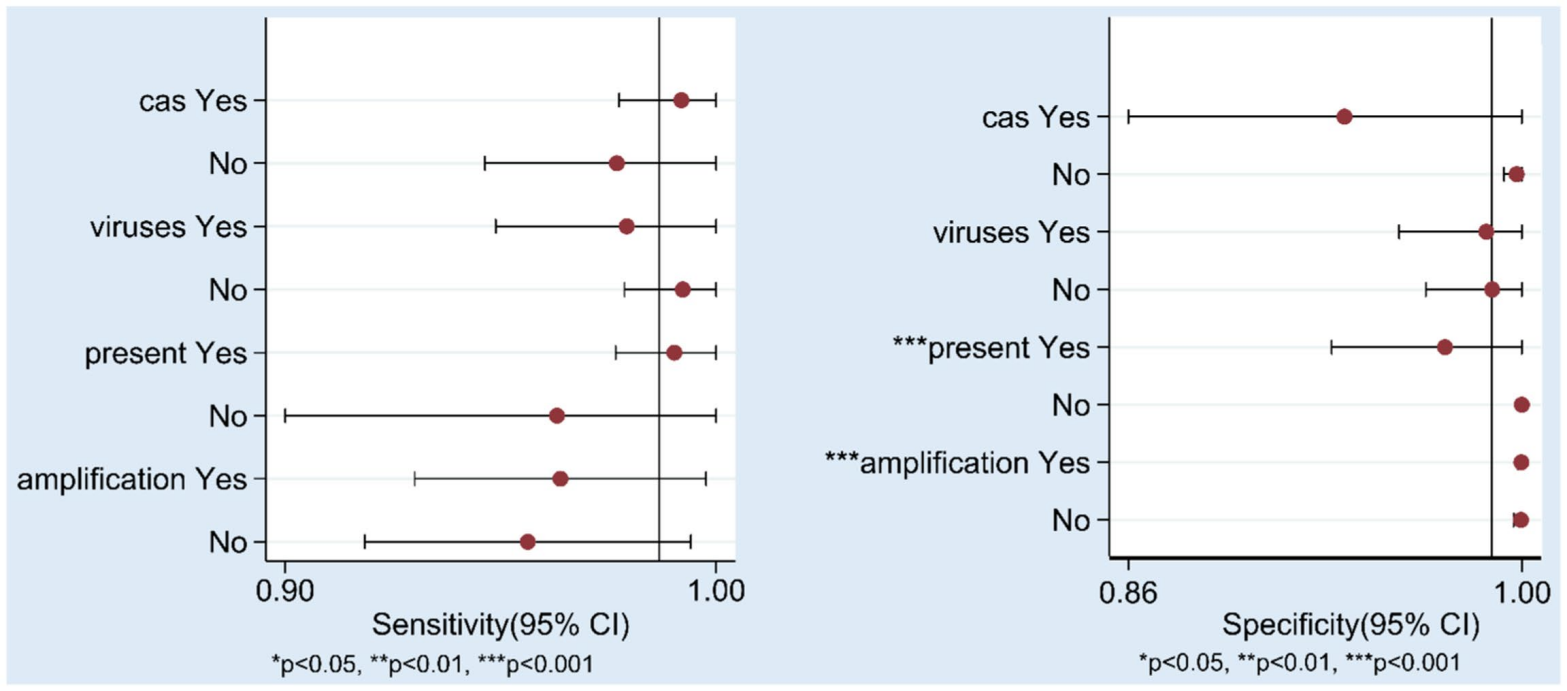
Results of Meta-regression. cas: CRISPR-Cas12/CRISPR-Cas13, virus: DNA/RNA viruses, present: fluorescence/test strip, amplification: PCR/RAA(RPA)/other amplification methods.

To explore the sources of heterogeneity, we conducted subgroup analyses. First, we classified the studies based on the type of Cas protein used in the established NAT methods: 10 studies used Cas12 protein, and 10 studies used Cas13 protein. For studies using the Cas12 protein, the pooled sensitivity was 0.98 (95% CI 0.93–0.99), the specificity was 1.00 (95% CI 0.54–1.00), and the area under the SROC curve was 1.00 (95% CI 0.99–1.00). For studies using the Cas13 protein, the pooled sensitivity was 0.99 (95% CI 0.94–1.00), the specificity was 0.95 (95% CI 0.79–0.99), the area under the SROC curve was 1.00 (95% CI 0.99–1.00), and the results are shown in the Supplementary Figures S2, S3 (A: cas12/ B: cas13). And the forest plot is shown in the Supplementary Figures S4, S5 (A: cas12/ B: cas13). Detailed results of individual and pooled sensitivity and specificity are presented in Table 2. The pooled accuracy data of the CRISPR-Cas12/13 for detecting hepatitis viruses is presented in Table 3. Due to the limited number of studies included, other variables, including the type of hepatitis virus detected (DNA or RNA viruses), the result presentation format (fluorescence, strip) and sample nucleic acid amplification methods (PCR, recombinase polymerase amplification, RPA, recombinase-aid amplification, RAA/other amplification methods) were analyzed using meta-regression, and the results are shown in the Figure 5. In the sensitivity analysis, none of these four factors were statistically significant for sensitivity. In terms of specificity, both the format of result presentation and amplification methods exhibited statistical significance. This indicates that there were statistically significant differences in specificity based on whether the results were presented using fluorescence or test strips, as well as based on the sample nucleic acid amplification methods. These two factors may have contributed to the heterogeneity of specificity to some extent.

**Table 2:** Individual and pooled sensitivity and specificity of CRISPR for detecting hepatitis virus.

Method and studies	TP	FN	Sensitivity (95% CI)	TN	FP	Specificity (95% CI)
Cas12 (*n* = 10)						
Chen et al. (2021)	72	0	1.00 (0.95–1.00)	42	0	1.00 (0.92–1.00)
Kham-Kjing et al. (2022)	189	11	0.94 (0.90–0.97)	60	0	1.00 (0.94–1.00)
Kham-Kjing et al. (2022)	189	11	0.94 (0.90–0.97)	60	0	1.00 (0.94–1.00)
Ding et al. (2021)	32	0	1.00 (0.89–1.00)	41	0	1.00 (0.91–1.00)
Ding et al. (2021)	32	0	1.00 (0.89–1.00)	41	0	1.00 (0.91–1.00)
Yue et al. (2021)	11	0	1.00 (0.72–1.00)	6	0	1.00 (0.54–1.00)
Ho et al. (2023)	8	0	1.00 (0.63–1.00)	9	0	1.00 (0.66–1.00)
Xu et al. (2024)	198	2	0.99 (0.96–1.00)	36	0	1.00 (0.90–1.00)
He et al. (2024)	47	4	0.92 (0.81–0.98)	25	0	1.00 (0.86–1.00)
Nguyen et al. (2023)	33	7	0.82 (0.67–0.93)	39	1	0.98 (0.87–1.00)
Pooled	811	35	0.98 (0.93–0.99)	359	1	1.00 (0.54–1.00)
Cas13 (*n* = 10)						
Tian et al. (2023b)	96	0	1.00 (0.96–1.00)	26	22	0.54 (0.39–0.69)
Tian et al. (2023b)	96	0	1.00 (0.96–1.00)	40	8	0.83 (0.70–0.93)
Tian et al. (2023a)	119	13	0.90 (0.84–0.95)	40	0	1.00 (0.91–1.00)
Tian et al. (2023a)	119	13	0.90 (0.84–0.95)	40	0	1.00 (0.91–1.00)
Li et al. (2023)	88	1	0.99 (0.94–1.00)	26	0	1.00 (0.87–1.00)
Li et al. (2023)	81	7	0.92 (0.84–0.97)	26	0	1.00 (0.87–1.00)
Zhang et al. (2022)	4	0	1.00 (0.40–1.00)	56	10	0.85 (0.74–0.92)
Zhang et al. (2022)	4	0	1.00 (0.40–1.00)	38	28	0.58 (0.45–0.70)
Wang et al. (2021)	106	0	1.00 (0.97–1.00)	196	10	0.95 (0.91–0.98)
Tian et al. (2022)	16	0	1.00 (0.79–1.00)	14	4	0.78 (0.52–0.94)
Pooled	729	34	0.99 (0.94–1.00)	502	82	0.54 (0.39–0.69)
Pooled	1,540	69	0.99 (0.95–1.00)	861	83	0.99 (0.93–1.00)

CI, confidence interval.

**Table 3:** Summary table of the diagnostic accuracy of CRISPR-Cas12/13 for detecting hepatitis virus.

	Sensitivity (95% CI)	Specificity (95% CI)	DOR (95% CI)	LRpos (95% CI)	LRneg (95% CI)	AUC (95% CI)
Cas12	0.98 (0.93–0.99)	1.00 (0.54–1.00)	352,189 (3.2–3.9 × 10^9^)	8488.7 (1.1–6.3 × 10^7^)	0.02 (0.01–0.08)	1.00 (0.99–1.00)
Cas13	0.99 (0.94–1.00)	0.95 (0.79–0.99)	3,308 (430–25,434)	18.1 (4.4–74.0)	0.01 (0.00–0.06)	1.00 (0.99–1.00)
Pooled	0.99 (0.95–1.00)	0.99 (0.93–1.00)	5,698 (1,049–30,958)	79.9 (14.2–449.1)	0.01 (0.00–0.05)	1.00 (0.99–1.00)

LRpos, positive likelihood ratio; LRneg, negative likelihood ratio; AUC, area under the curve.

## 4 Discussion

NAT results are widely accepted as a key indicator of hepatitis virus detection. However, the commonly employed qPCR for NAT faces several limitations that hinder large-scale screening, post-treatment monitoring, and utilization in regions with limited medical resources (Peeling et al., 2017). The CRISPR-Cas system, with its unique collateral cleavage activity of Cas proteins and efficient signal amplification mechanisms, offers a novel approach for ultra-sensitive and portable NAT.

In this systematic review and meta-analysis, we evaluated the accuracy of the CRISPR-Cas system in detecting hepatitis viruses. Our findings revealed a pooled sensitivity and specificity of 0.99 (95% CI: 0.95–1.00) and 0.99 (95% CI: 0.93–1.00), respectively, for hepatitis virus detection using the CRISPR-Cas system. The SROC curve demonstrated an AUC of 1.00 (95% CI: 0.99–1.00), indicating excellent diagnostic performance.

The *I*^2^ tests for sensitivity and specificity yielded values of 77.86 (95% CI: 68.29–87.43) and 94.90 (95% CI: 93.46–96.34), respectively, indicating substantial heterogeneity among the studies. To explore the sources of this heterogeneity, we stratified the included studies into Cas12 and Cas13 protein groups and constructed forest plots and SROC curves for each group. The results showed that for the Cas12 group, the pooled sensitivity was 0.98 (95% CI: 0.93–0.99) with an *I*^2^ test of 87.15 (95% CI: 80.45–93.85), while the pooled specificity was 1.00 (95% CI: 0.54–1.00) with an *I*^2^ test of 49.74 (95% CI: 13.33–86.15). For the Cas13 group, the pooled sensitivity was 0.99 (95% CI: 0.94–1.00) with an *I*^2^ test of 76.75 (95% CI: 62.53–90.97), and the pooled specificity was 0.95 (95% CI: 0.79–0.99) with an *I*^2^ test of 92.93 (95% CI: 89.83–96.03). Although a slight reduction in *I*^2^ values was observed after stratification, the differences were not statistically significant, suggesting that the use of different Cas proteins was not the primary source of heterogeneity. To further investigate the sources of heterogeneity, we performed meta-regression analysis, which revealed no statistically significant differences in sensitivity or specificity between the different Cas proteins, consistent with the subgroup analysis. It is noteworthy that there exist statistical differences in specificity between research on detecting DNA viruses and RNA viruses. This may be attributed to the fact that among the five common types of hepatitis viruses, only HBV is a DNA virus. Consequently, researchers have conducted more extensive studies on HBV, which has enhanced the accuracy of detection methods related to DNA viruses among hepatitis viruses. Furthermore, specific differences were also observed among studies based on the presentation of test results, which could be ascribed to the subjective nature of lateral flow analysis evaluation compared to fluorescence-based tests. Due to the existence of multiple genotypes within the same hepatitis virus, and a study has indicated the occurrence of recombination among different HBV genotypes (Liu et al., 2018). Consequently, the use of detection methods tailored to specific genotypes may be one of the reasons underlying the heterogeneity observed among various studies. Furthermore, the method of nucleic acid extraction from samples directly determines the test results, and differing nucleic acid extraction methods may also be one of the sources of heterogeneity among various studies. However, given the limited data included in this study, further research is still required to support these hypothesis.

It is important to note that this systematic review and meta-analysis relied on qPCR as the gold standard. However, the gold standard may not always accurately reflect the true sample status. For example, in the study of Zhang et al. (2022), a CRISPR-Cas-based test for detecting HBV cccDNA was developed. Compared to qPCR, the new method has a higher false positive rate, but the results are closer to those of digital drop PCR (ddPCR), which may indicate greater sensitivity of the CRISPR-Cas system. Similarly, Wang et al. (2021) reported 10 “false positive” results inconsistent with qPCR, but confirmed as low viral load positive by ddPCR, further validating the potential of CRISPR-Cas to outperform qPCR in sensitivity, which may have led to an underestimation of the specificity of the CRISPR-Cas system for detecting hepatitis virus.

## 5 Limitations

This study is subject to several limitations. Firstly, there exists significant heterogeneity among the included studies, which we addressed by adopting a random-effects model to mitigate its impact. Additionally, we attempted to conduct subgroup analyses to explore the sources of heterogeneity. However, due to an inadequate number of studies, we were unable to further analyze and draw definitive conclusions.

Secondly, a potential source of bias arises from the nature of the research paradigm employed. Since all the included studies adhered to a pattern of establishing a method followed by clinical sample validation, there is a tendency for authors to preferentially report positive findings. Conversely, studies yielding negative results may be less likely to be published, as researchers may lack the motivation to document them (Siddaway et al., 2019). This publication bias could inflate the apparent accuracy of the results beyond what is actually observed in practice. We also confirmed the existence of publication bias by drawing funnel plot (Supplementary Figure S2).

Thirdly, qPCR, which serves as the gold standard for comparison, is not without its own limitations. Firstly, qPCR may not always accurately reflect the true state of the samples due to various factors such as sample preparation, primer design, and amplification efficiency. Secondly, the lack of complete standardization of qPCR techniques across different laboratories hinders the direct comparability of results. This variability in techniques can introduce additional uncertainty into the interpretation of our study (Rizzetto and Hamid, 2021).

Finally, the novelty of CRISPR-Cas system for hepatitis virus detection is a factor that necessitates caution in interpreting our results. Given that this technology has only recently gained traction for this application, all the included studies in our analysis were published within the last 5 years. As such, our conclusions are based on a relatively limited body of evidence, and further research is warranted to refine this methodology and strengthen the support for our findings. More comprehensive and long-term studies are needed to fully evaluate the potential of CRISPR-Cas system in hepatitis virus detection.

## 6 Conclusion

The detection of hepatitis viruses remains a pivotal task within the realm of liver diseases and infectious diseases. This systematic review and meta-analysis, for the first time, presents diagnostic accuracy data for the utilization of the CRISPR-Cas system in detecting hepatitis viruses. However, due to the notable heterogeneity observed in the current studies, our findings necessitate further validation with more high-quality research data. Overall, the CRISPR-Cas system, with its excellent diagnostic accuracy, shows great potential in the identification of various hepatitis viruses and is expected to become a powerful alternative to traditional detection methods, leading a new chapter in future hepatitis virus diagnostic technology.

## Data Availability

The original contributions presented in the study are included in the article/Supplementary material, further inquiries can be directed to the corresponding author.

## Supplementary material

SUPPLEMENTARY FIGURE S1 Funnel plot for included studies.

SUPPLEMENTARY FIGURE S2 The SROC curves of the CRISPR-Cas12/13 system in diagnosis of hepatitis viruses. **(A)** CRISPR-Cas12. **(B)** CRISPR-Cas13.

SUPPLEMENTARY FIGURE S3 Forest plots of the pooled sensitivity and specificity for CRISPR-Cas12/13 system in diagnosis of hepatitis viruses. **(A)** CRISPR-Cas12. **(B)** CRISPR-Cas13. Image 1: Supplementary Figure S1 Image 2: Supplementary Figure S2 Image 3: Supplementary Figure S3 Image 4: Supplementary Figure S4 Image 5: Supplementary Figure S5
