# Supplementary figures and images for: CRISPR-Cas for hepatitis virus: a systematic review and meta-analysis of diagnostic test accuracy studies
Source: Front Microbiol. 2025 Mar 3;16:1509890. doi: 10.3389/fmicb.2025.1509890 (PMC11912011; doi:10.3389/fmicb.2025.1509890)

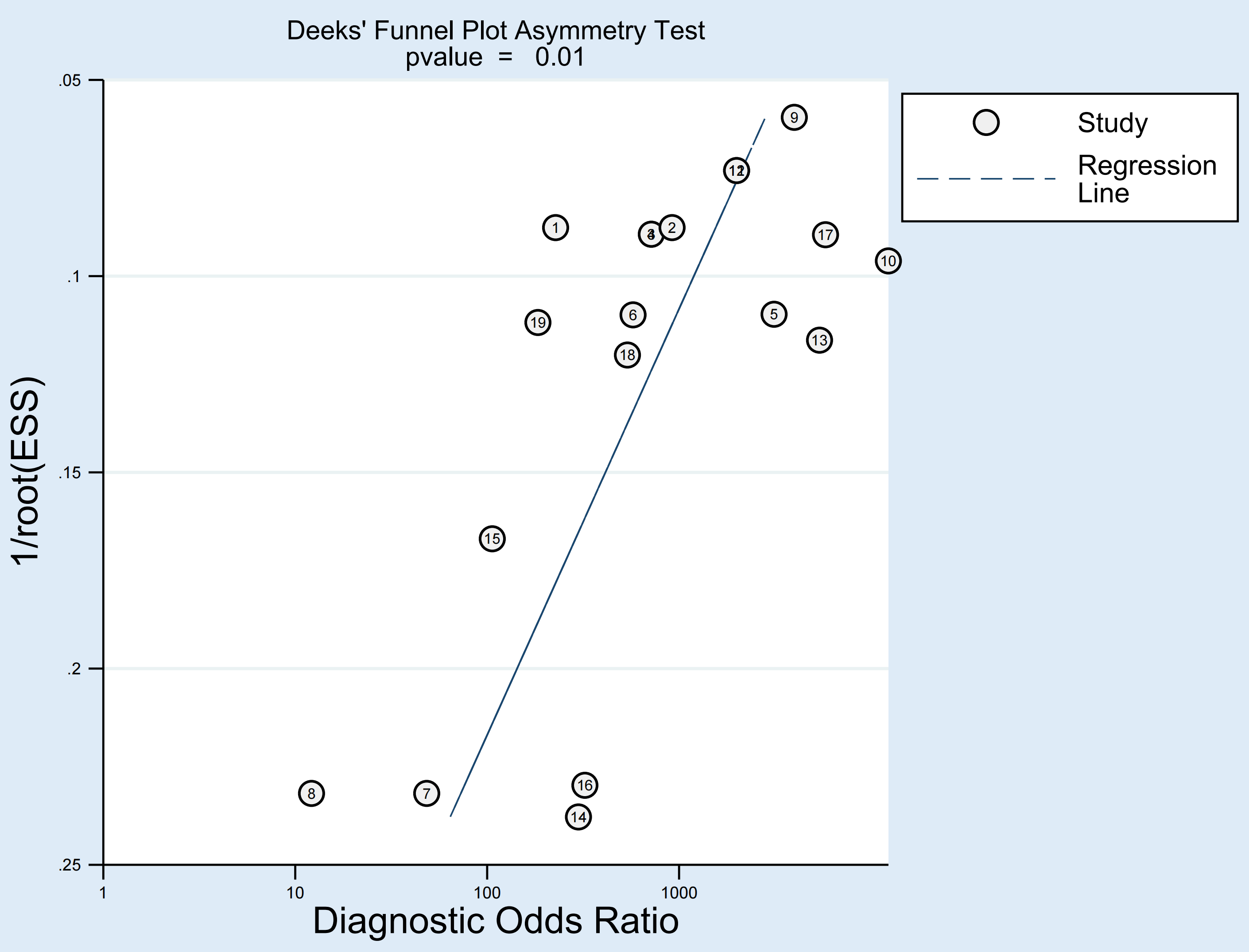

Supplement: SUPPLEMENTARY FIGURE S1 — Funnel plot for included studies. [file Image_1.png]

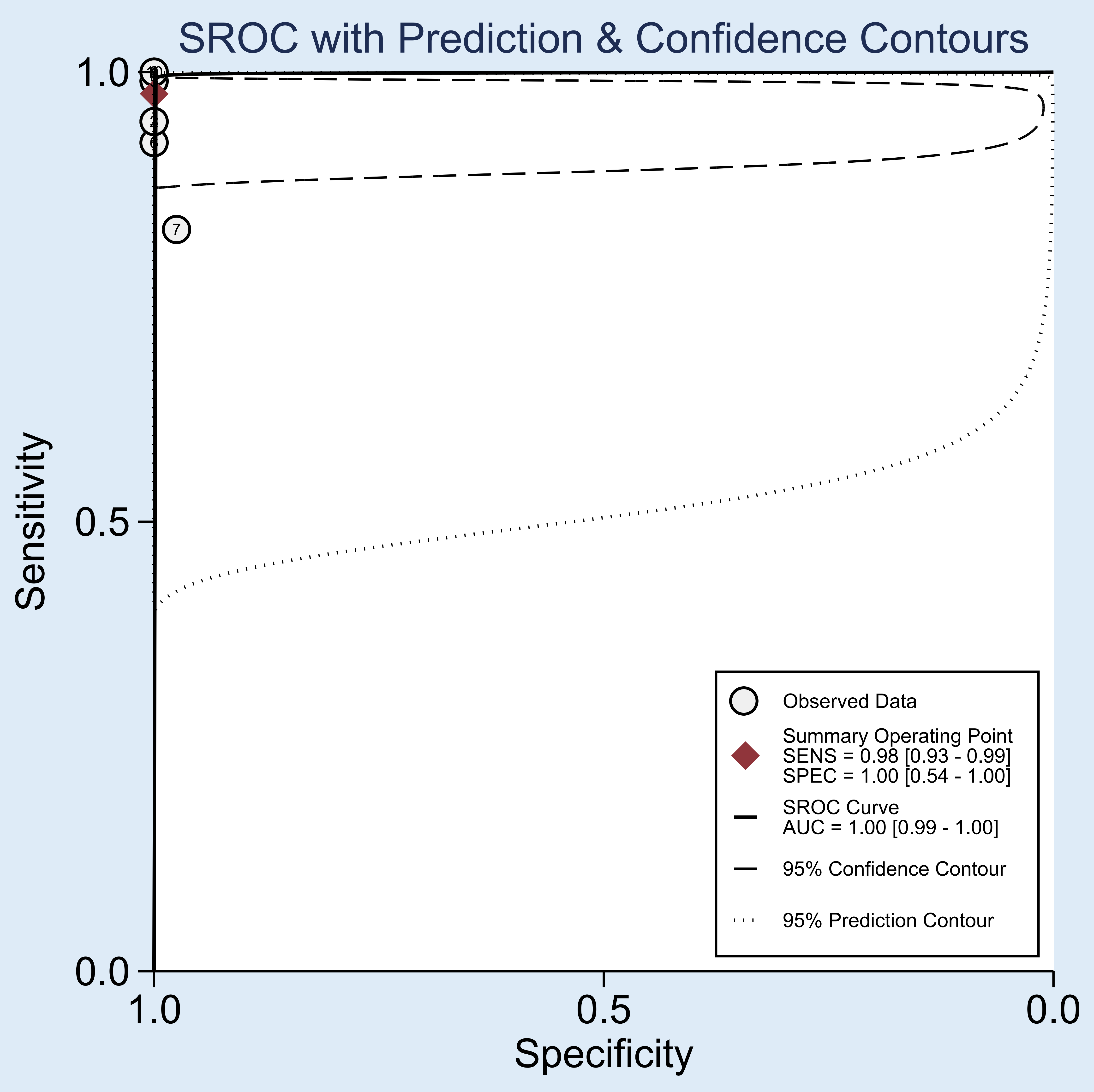

Supplement: SUPPLEMENTARY FIGURE S2 — The SROC curves of the CRISPR-Cas12/13 system in diagnosis of hepatitis viruses. (A) CRISPR-Cas12. (B) CRISPR-Cas13. [file Image_2.png]

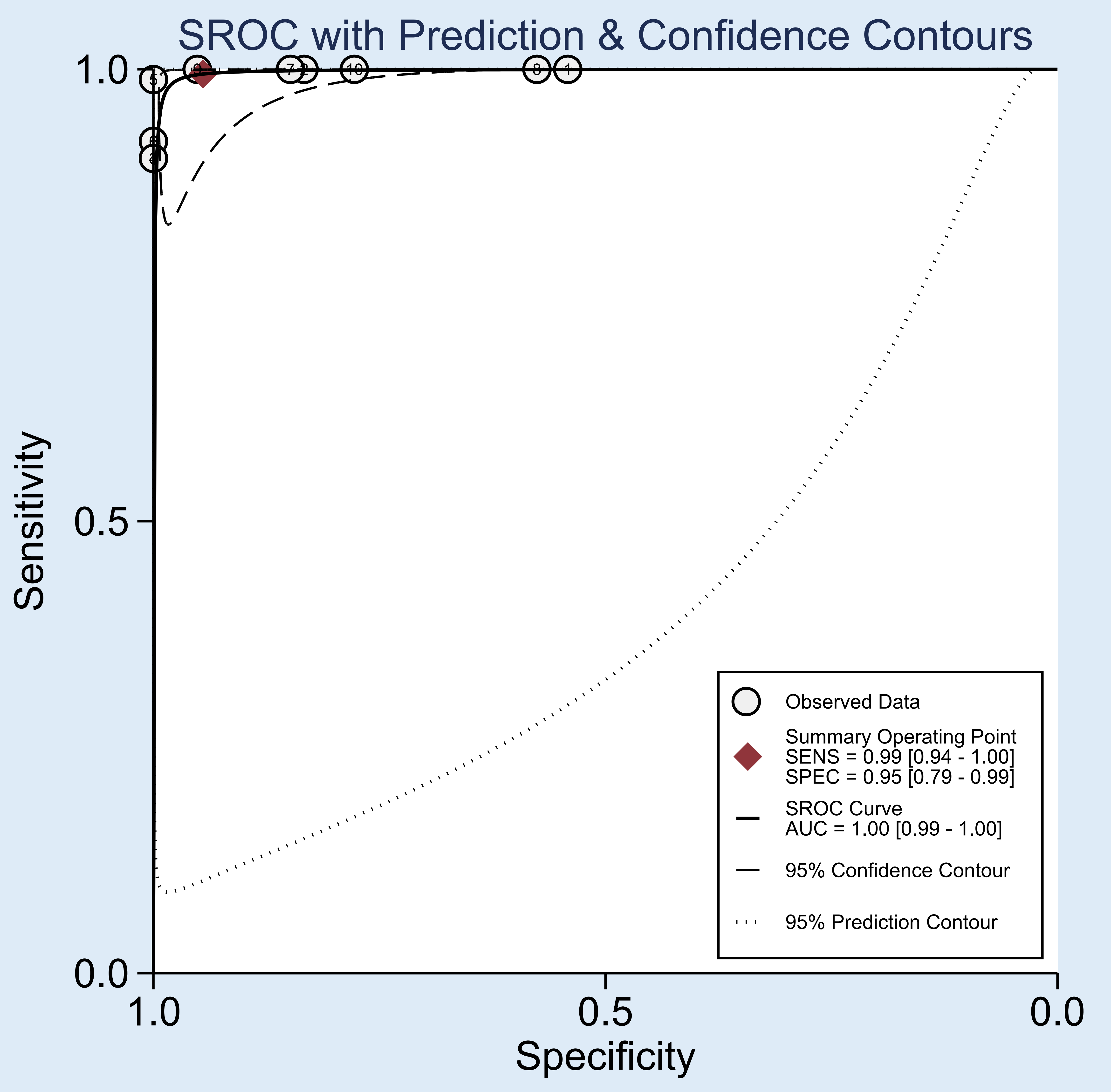

Supplement: SUPPLEMENTARY FIGURE S3 — Forest plots of the pooled sensitivity and specificity for CRISPR-Cas12/13 system in diagnosis of hepatitis viruses. (A) CRISPR-Cas12. (B) CRISPR-Cas13. Image 1: Supplementary Figure S1 Image 2: Supplementary Figure S2 Image 3: Supplementary Figure S3 Image 4: Supplementary Figure S4 Image 5: Supplementary Figure S5 [file Image_3.png]

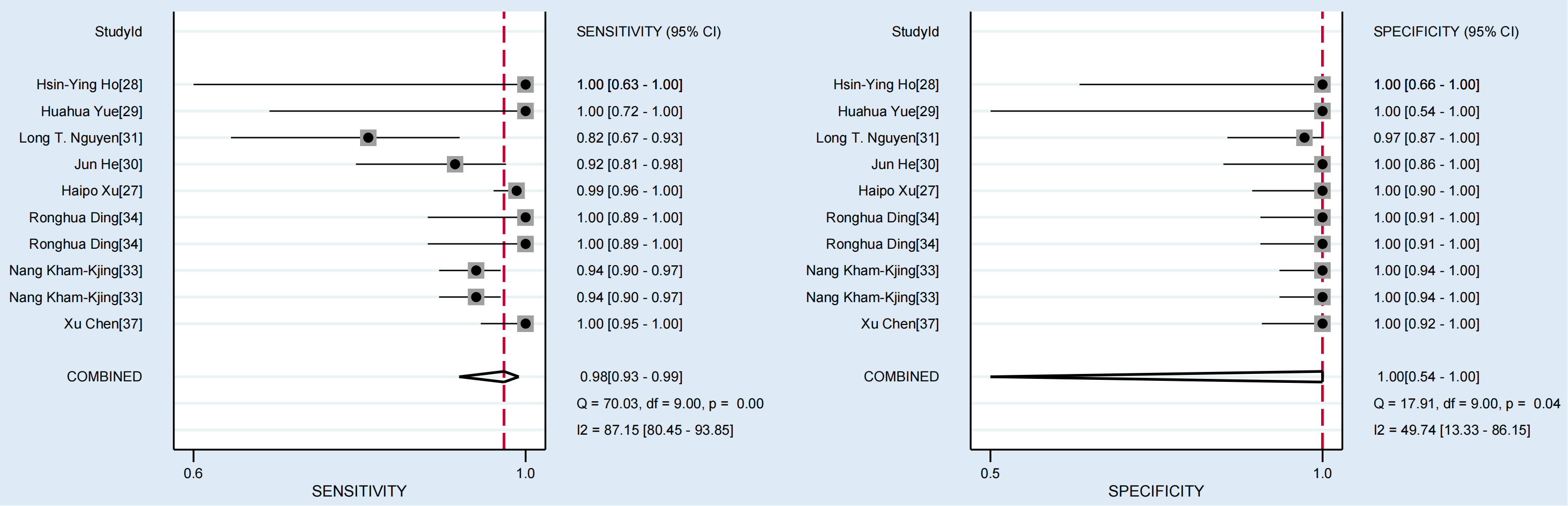

Supplement: Supplementary file 4 [file Image_4.tif]

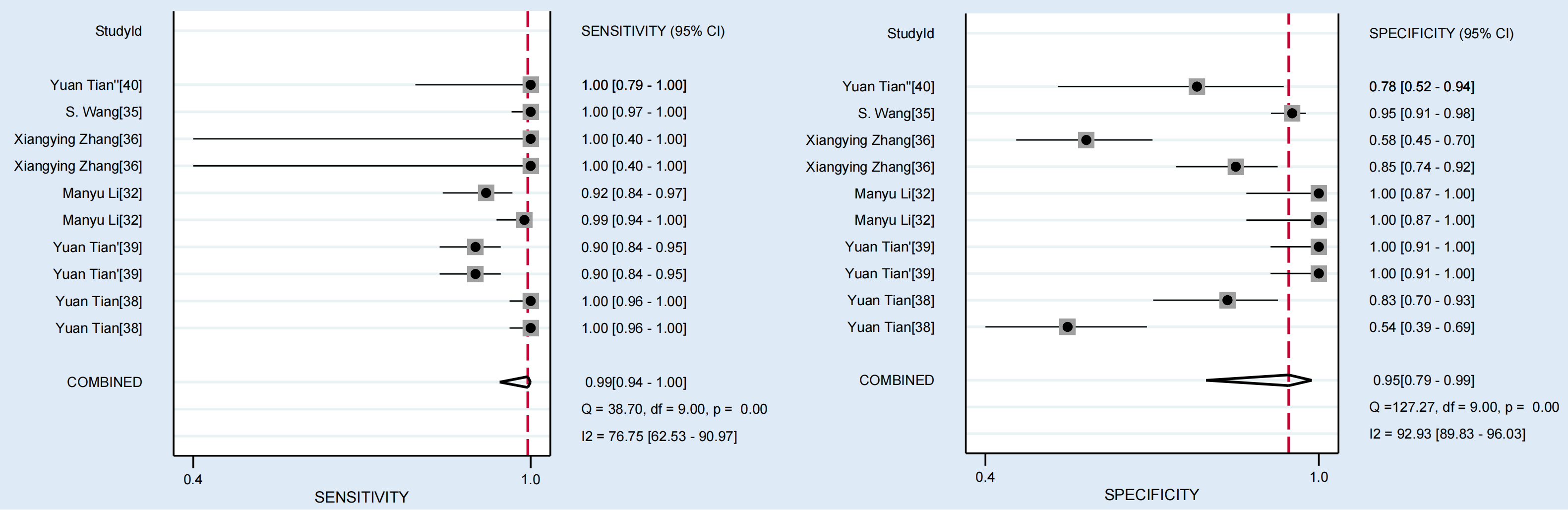

Supplement: Supplementary file 5 [file Image_5.tif]
