# Supplementary material for: CRISPR-Cas for hepatitis virus: a systematic review and meta-analysis of diagnostic test accuracy studies
Source: Front Microbiol. 2025 Mar 3;16:1509890. doi: 10.3389/fmicb.2025.1509890 (PMC11912011; doi:10.3389/fmicb.2025.1509890)
Supplement: Supplementary file 6 [file Supplementary_file_1.docx]

**Supplementary File**. Search Strategy

**Search Database:** PubMed

**Publication date:** January 1, 1900, to August 1, 2024.

**Search Strategy:** ((HAV) OR (HBV) OR (HCV) OR (HDV) OR (HEV) OR (hepatitis virus) OR (hepatitis A) OR (hepatitis B) OR (hepatitis C) OR (hepatitis D) OR (hepatitis E)) AND (CRISPR) AND ("1900/1/1"[Date - Publication] : "2024/8/1"[Date - Publication])

**Search Database:** Web of science

**Publication date:** January 1, 1900, to August 1, 2024.

**Search Strategy:** 1: TI=(hepatitis virus) OR TI=(HAV) OR TI=(hepatitis A) OR TI=(HBV) OR TI=(hepatitis B) OR TI=(HCV) OR TI=(hepatitis C) OR TI=(HDV) OR TI=(hepatitis D) OR TI=(HEV) OR TI=(hepatitis E) and Preprint Citation Index (Exclude – Database) Timespan: 1900-01-01 to 2024-08-01 Results: 237650

2: TI=(CRISPR) OR TI=(cas) and Preprint Citation Index (Exclude – Database) Timespan: 1900-01-01 to 2024-08-01 Results: 85164

3: #2 AND #1 and Preprint Citation Index (Exclude – Database) Timespan: 1900-01-01 to 2024-08-01 Results: 397

**Search Database:** CNKI

**Publication date:** January 1, 1900, to August 1, 2024.

**Search Strategy:** 1: TI%=('CRISPR' + 'cas') AND TI%=('HAV' + 'HBV' + 'HCV' + 'HDV' + 'HEV' + '肝炎病毒')
